# Surgical procedures for treatment of adult acquired flatfoot deformity: a network meta-analysis

**DOI:** 10.1186/s13018-019-1094-0

**Published:** 2019-02-21

**Authors:** Xu Tao, Wan Chen, Kanglai Tang

**Affiliations:** Department of Orthopedic Surgery, Southwest Hospital, Army Medical University, Chongqing, 400038 China

**Keywords:** Adult acquired flatfoot deformity, Surgical treatment, Meta-analysis

## Abstract

**Background:**

Adult acquired flatfoot deformity (AAFD) represents a spectrum of deformities affecting the foot and the ankle. The optimal management of AAFD remains controversial. We evaluated the efficacy of surgical treatments of AAFD using both direct and indirect evidences.

**Methods:**

We searched PubMed, EmBase, and the Cochrane Library to identify eligible studies conducted through November 2018. To compare different surgical strategies, we performed a network meta-analysis. A traditional meta-analysis using a random-effects model was used to evaluate the pooled outcome.

**Results:**

A total of 21 studies including 498 patients were collected and analyzed. Network meta-analysis results based on lateral angle talocalcaneal-calcaneal pitch (LAT-CP) indicated that medial displacement calcaneal osteotomy (MDCO) has the highest probability to be the best course of AAFD treatment. However, analyses based on anteroposterior talo-first metatarsal (AP-TMT1) and lateral angle talocalcaneal talo-first metatarsal (LAT-TMT1) suggested that lateral column lengthening (LCL) was the best treatment, while those based on lateral angle talocalcaneal-arch height, anteroposterior talocalcaneal (AP-TC), lateral angle talocalcaneal-talocalcaneal (LAT-TC), anteroposterior-talonavicular coverage (AP-TNC), talonavicular coverage (TNC), and the American Orthopedic Foot and Ankle Society (AOFAS) indicated triple arthrodesis (TAO) as the best treatment. Moreover, double arthrodesis (DAO) provided the best treatment effect on the function score. Furthermore, according to traditional meta-analysis, the summary of standardized mean differences (SMD) indicated that the surgical interventions are associated with significant improvements in LAT-CP (SMD − 1.78), LAT-arch height (SMD − 4.95), AOFAS (SMD − 5.24), AP-TMT1 (SMD 2.45), LAT-TMT1 (SMD 1.97), AP-TC (SMD 3.05), LAT-TC (SMD 2.20), AP-TNC (SMD 2.07), TNC (SMD 1.70), and function score (SMD 0.95).

**Conclusions:**

Our findings indicated that MDCO, LCL, TAO, or DAO might be the best surgical approaches for AAFD treatment. Furthermore, patients who received surgical interventions had significant improvements in symptoms and function.

**Electronic supplementary material:**

The online version of this article (10.1186/s13018-019-1094-0) contains supplementary material, which is available to authorized users.

## Background

Adult acquired flatfoot deformity (AAFD) is a degenerative disease characterized by pathological changes in the tibialis posterior tendon, spring ligament complex, deltoid ligament, and other ligaments of the hindfoot [1]. This is divided into four stages. Stage I is characterized by pain and swelling in the tibialis posterior tendon but have normal alignment. Stage II is characterized by flexible deformities in the foot and can also include the hindfoot valgus, varying arch height, and degrees of abduction and supination of the forefoot. Stage III is similar to stage II, but the deformities are more severe and are rigid rather than flexible. Lastly, stage IV is characterized by fixed deformities and valgus tilting of the talus [2–4]. The pathogenesis of AAFD still remains unclear. The possible pathogenic factors include varying stresses on surrounding joints and weakening of the dynamic and static ligamentous restraints of the hindfoot and midfoot [1].

The treatment of AAFD involves slowing down the progression of the disease. Surgical techniques, such as soft tissue with medial or lateral column procedures, are widely used to avoid the progression of these fixed deformities [1]. According to a previous study, the medial surgical approach permitted fusion without the development of non-union and provided a significant correction of the fixed deformities. However, there are limited studies that compared the efficacy among these surgical techniques, and the best approach for treating AAFD still remains controversial.

Currently, the common surgical procedures included medial displacement calcaneal osteotomy (MDCO), lateral column lengthening (LCL), modified triple arthrodesis (MTA), and flexor digitorum longus (FDL) transfer. These strategies are widely used for mild deformities with flexibility, whereas no quantitative indexes are constructed for operative indication. Previous studies have showed that these interventions can slow down the progression and relieve the clinical symptoms of AAFD, but a clear comparison of the efficacy among these surgical procedures is still needed [5, 6]. Hence, this network meta-analysis aimed to use both direct and indirect evidences to evaluate the comparative efficacy of surgical procedures in AAFD patients.

## Methods

### Data sources, search strategy, and selection criteria

This review was conducted and reported according to the Preferred Reporting Items for Systematic Reviews and Meta-Analysis Statement, 2009 (Additional file 1: PRISMA Checklist) [7].

We performed systematic searches in PubMed, EmBase, and the Cochrane Library for relevant literature that is published on or before November 2018. The following terms were used separately in the search: “posterior tibial tendon dysfunction,” “pes planus,” “adult acquired flatfoot deformity,” “midfoot abduction,” “posterior tibial tendon,” “pes planovalgus,” “posterior tibial tendon insufficiency,” “flatfoot staging,” “flatfoot treatment,” “foot orthoses,” and “surgical”. The resulting titles and abstracts of the primary collections were browsed. To identify additional candidate studies, the reference lists of the included studies and reviews were also evaluated.

The literature search was performed by two independent authors, and any inconsistencies between them were settled by group discussion until a consensus was reached. A study was considered eligible for inclusion if the following criteria were met: (1) the trials that investigated surgical procedures for the treatment of AAFD patients and (2) the authors’ outcome reports included lateral angle talocalcaneal-calcaneal pitch (LAT-CP), anteroposterior talo-first metatarsal (AP-TMT1), lateral angle talocalcaneal talo-first metatarsal (LAT-TMT1), LAT-arch height, anteroposterior talocalcaneal (AP-TC), lateral angle talocalcaneal-talocalcaneal (LAT-TC), anteroposterior-talonavicular coverage (AP-TNC), TNC, the American Orthopedic Foot and Ankle Society (AOFAS), and function score. Cohorts, case controls, case series, reviews, and editorials were excluded due to uncontrolled confounders.

### Data collection and quality assessment

Two reviewers independently extracted all the data, and disagreements were resolved in consultation with third-party investigators. The following information was extracted from the included articles: first author, publication year, country, prospective or retrospective design, age, follow-up duration, disease type, surgery type, reported outcomes, and number of patients. The investigated outcomes from radiography examination included LAT-CP, AP-TMT1, LAT-TMT1, LAT-arch height, AP-TC, LAT-TC, AP-TNC, TNC, AOFAS, and function score. Two reviewers independently assessed the quality of the included studies according to the Jadad scale in the following five domains: randomization (1 or 0), concealment of the treatment allocation (1 or 0), blinding (1 or 0), completeness of follow-up (1 or 0), and the use of intention-to-treat analysis (1 or 0). Based on these results, the studies were given scores with a previously developed scoring system for quality assessment that ranged from 1 to 5 [8].

### Statistical analysis

For traditional meta-analyses, we used the inverse variance method to pool the continuous data. The results are presented as standardized mean difference (SMD) with 95% confidence intervals (95%CIs). In our network meta-analysis, we used a random-effect network meta-analysis for mixed multiple treatment comparisons, which fully preserves the within-trial randomized treatment comparisons of each trial [9].

The *I*^2^ statistic was calculated to evaluate the extent of variability that was attributable to statistical heterogeneity between the trials. In the absence of statistical heterogeneity (*I*^2^ < 50%), we used a fixed-effects model; otherwise, we used a random-effects model in a traditional meta-analysis [10, 11]. Consistency within every closed triangle or quadratic loop was investigated using a loop-specific approach in our network meta-analysis. During analysis, inconsistency factors and their 95%CIs were used to determine their compatibility with zero [12]. To rank the treatments for an outcome, we used surface under the cumulative ranking (SUCRA) probabilities [13]. A “comparison-adjusted” funnel plot was used to assess the presence of small-study effects in our network meta-analysis [14]. All tests were two-tailed, and *p* value of less than 0.05 was deemed to be statistically significant. We analyzed the data using STATA software (version 10.0).

## Results

**Fig. 1:**
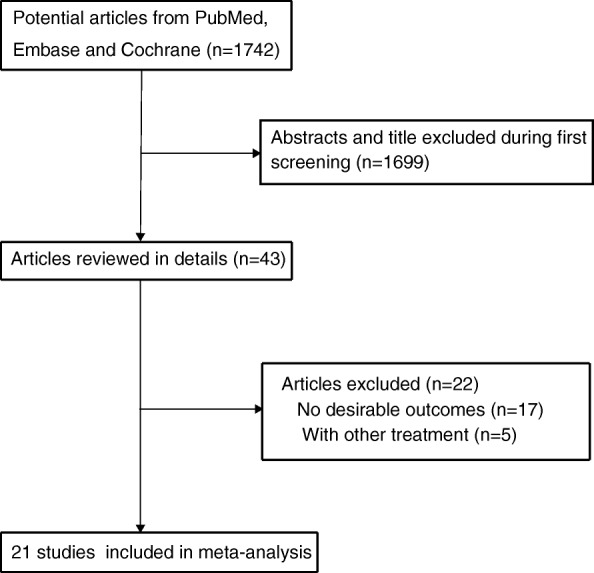
Flow diagram showing the study selection process

Our initial search produced 1798 unique results. Of these, we collected 21 trials that met our study criteria, which included 498 patients [15–35] (Fig. 1). After reviewing the full texts, the reasons for the exclusion of literature included other intervention interferences, other similar diseases, and lack of desired outcome assessment. The general characteristics of the included studies are presented in Table 1. Of the included studies, 10 were conducted in the US, 5 in Europe, 4 in China, 1 in Japan, and 1 in Egypt. Study quality was evaluated using the Jadad scale (Table 1). Overall, 8 studies had a score of 2, 10 studies had a score of 1, and the remaining 3 studies had a score of 0.

**Table 1:** Baseline characteristic of studies included in the systematic review and meta-analysis

Study	Publication year	Country	Sample size	Mean age	Follow-up	Disease status	Surgery type	Jadad score
Mark S	1995	America	18	54.0	(12–16) months	AAFD secondary to PTTI	MDCO, FDL, MTCP	1
Beat Hintermann	1999	Switzerland	19	52.9	35.8 (12–37) months	PTTI	LCL, medial soft tissue reconstruction	2
Charles M	1999	America	10	48.7	35 (8–60) months	Stage II/III PTTD	Evans LCL,TN fusion	1
Alan R	2000	America	24	53.7	27 (13–51) months	PTTD and AAFD	PCDO, FDL, ATL	1
G. James Sammarco	2001	America	17	60.0	17 (11–24) months	Stage II PTTD	MDCO,FHL	2
Gregory P	2001	America	26	54.0	31.8 (12–70) months	Stage II PTTD	FDL, MDCO	1
Amir H.	2002	America	23	56.0	35 (24–51) months	Stage II PTTD	FDL, calcaneal osteotomy	2
G.A.	2003	Netherlands	14	62.0	42 (6–78) months	Stage II PTTD	CC bone-block arthrodesis, medial soft tissue reconstruction	2
Arie van der Krans	2006	Netherlands	20	55.0	25.3 (13–39) months	Acquired flatfoot and PTDLS	LCL, ATL, FDL	2
Patrick M.	2007	America	40	46.5	30.5 (12–96) months	AAFD	LCL, MTO, FDL, GR, TMT	2
Kang-lai Tang	2010	China	10	41.8	13.2 (6–21) months	Stage II and IIB AAFD	Osteotomy of subtalar joint, talonavicular joint, and calcaneocuboid joint	1
Rémi Philippot	2010	France	14	59.4	20.6 (12–54) months	Pes planovalgus	Subtalar and talonavicular joint arthrodeses	0
Guus A.	2010	Netherlands	33	57.8	29.2 (6–78) months	Flexible acquired flatfoot	Lengthening calcaneus osteotomy, CCJ distraction arthrodesis	1
Yehia Basioni	2011	Egypt	14	23.0	22 (13–34) months	Flexible adult acquired flatfoot	Evans osteotomy and posterior calcaneal displacement osteotomy	1
Thomas H.	2011	America	41	52.0	9.6 (3–43) months	AAFD	Medial column arthrodesis, MDCO	2
Hong-hui Cao	2012	China	33	30.1	23.0 (6–54) months	Flatfoot related with accessory navicular	Reconstruction of posterior tibial tendon	1
Hisateru Niki	2012	Japan	25	55.2	5.6 (2.6–10.2) years	Stage II PTTD	MDCO, FDL	0
Michael Iossi	2013	America	68	55.0	9 (2–30) months	AAFD	FDL, MDCO, LCL	2
Siddhant K.	2013	America	21	68.0	13 (6–44) months	Stage II PTTD	Fusions of the subtalar, talonavicular, and first tarsometatarsal joints	1
Hong-hui Cao	2014	China	16	41.5	12 months	Flexible flatfoot	MDCO, reconstruction of the PTT	1
Guangrong Yu	2014	China	12	53.3	19.4 (13–30) months	Flatfoot deformity with pes valgus	Subtalar and talonavicular joints arthrodesis through a single medial incision approach	0

Traditional meta-analysis was used to evaluate the effect of surgical intervention for AAFD, and a summary of the outcomes is listed in Table 2. Overall, we noted that surgical intervention was associated with lower levels of LAT-CP (SMD − 1.78; 95%CI − 2.57 to − 0.99; *p* < 0.001; *I*^2^ 92.4%), LAT-arch height (SMD − 4.95; 95%CI − 5.69 to − 4.20; *p* < 0.001; *I*^2^ 0.0%), and AOFAS (SMD − 5.24; 95%CI − 6.98 to − 3.49; *p* < 0.001; *I*^2^ 90.7%). Furthermore, surgical treatments were associated with AAFD patients with higher levels of AP-TMT1 (SMD 2.45; 95%CI 1.74 to 3.17; *p* < 0.001; *I*^2^ 91.5%), LAT-TMT1 (SMD 1.97; 95%CI 1.18 to 2.77; *p* < 0.001; *I*^2^ 95.4%), AP-TC (SMD 3.05; 95%CI 1.37 to 4.73; *p* < 0.001; *I*^2^ 96.2%), LAT-TC (SMD 2.20; 95%CI 0.98 to 3.42; *p* < 0.001; *I*^2^ 94.8%), AP-TNC (SMD 2.07; 95%CI 1.04 to 3.09; *p* < 0.001; *I*^2^ 92.1%), TNC (SMD 1.70; 95%CI 1.00 to 2.40; *p* < 0.001; *I*^2^ 83.1%), and function score (SMD 0.95; 95%CI 0.24 to 1.67; *p* = 0.009; *I*^2^ 90.7%). Although substantial heterogeneity was detected for most of the outcomes, the conclusions were not affected by the sequential exclusion of any single study.

**Table 2:** Summary of the standard mean difference of all outcomes assessed by using traditional meta-analysis

Outcomes	SMD	95% CI	*p* value	Heterogeneity (%)	*p* value for heterogeneity
LAT-CP	− 1.78	− 2.57 to − 0.99	< 0.001	92.4	< 0.001
AP-TMT1	2.45	1.74 to 3.17	< 0.001	91.5	< 0.001
LAT-TMT1	1.97	1.18 to 2.77	< 0.001	95.4	< 0.001
LAT-arch height	− 4.95	− 5.69 to − 4.20	< 0.001	0.0	0.398
AP-TC	3.05	1.37 to 4.73	< 0.001	96.2	< 0.001
LAT-TC	2.20	0.98 to 3.42	< 0.001	94.8	< 0.001
AP-TNC	2.07	1.04 to 3.09	< 0.001	92.1	< 0.001
TNC	1.70	1.00 to 2.40	< 0.001	83.1	< 0.001
AOFAS	− 5.24	− 6.98 to − 3.49	< 0.001	90.7	< 0.001
Function score	0.95	0.24 to 1.67	0.009	90.7	< 0.001

**Fig. 2:**
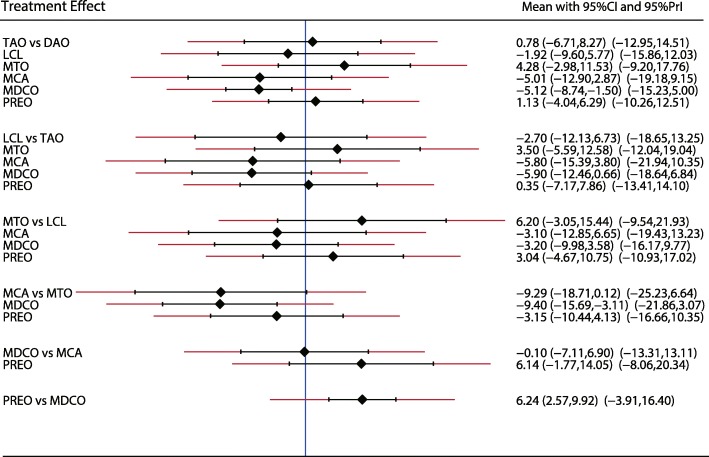
Comparisons of different surgical interventions for AAFD

The eligible LAT-CP comparisons in our network meta-analysis are predominantly pairwise comparisons of different surgical interventions for AAFD patients (Fig. 2). The modes in Fig. 2 are weighted according to the number of patients that received each surgical mode, and the edges are weighted according to the mean control group for all comparisons versus the corresponding preoperative values. The MDCO versus preoperative treatment demonstrated the highest contribution for the entire network meta-analysis. The LAT-CP results showed that the LAT-CP values following TAO (SMD − 6.24; 95%CI − 11.59 to − 0.89) or MDCO (SMD − 9.31; 95%CI − 18.59 to − 0.12) are significantly lower than the corresponding preoperative values, but none of the other comparisons revealed significant differences.

**Fig. 3:**
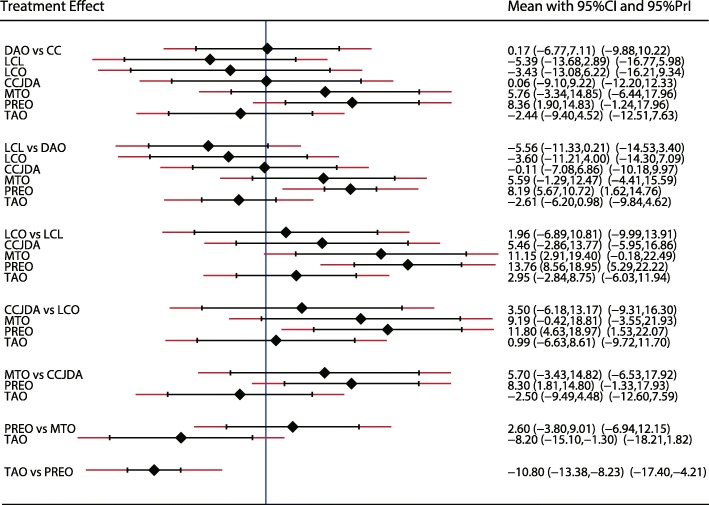
The effect of different treatment strategies based on changes in AP-TMT1 values

**Fig. 4:**
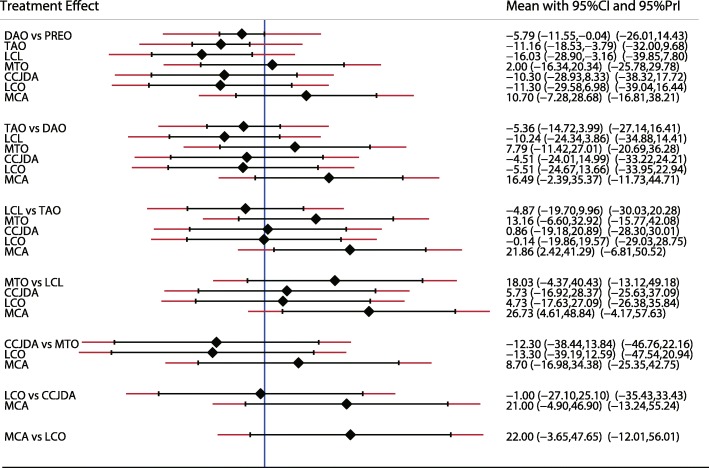
The effect of different treatment strategies based on changes in LAT-TMT1 values

**Fig. 5:**
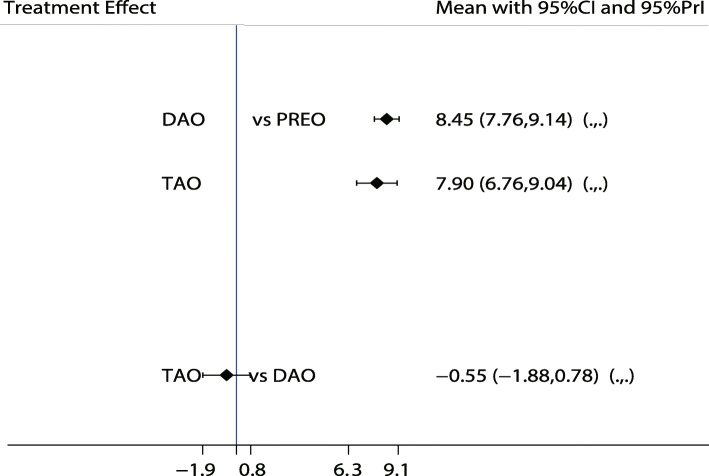
The effect of different treatment strategies based on changes in the LAT-Arch height

**Fig. 6:**
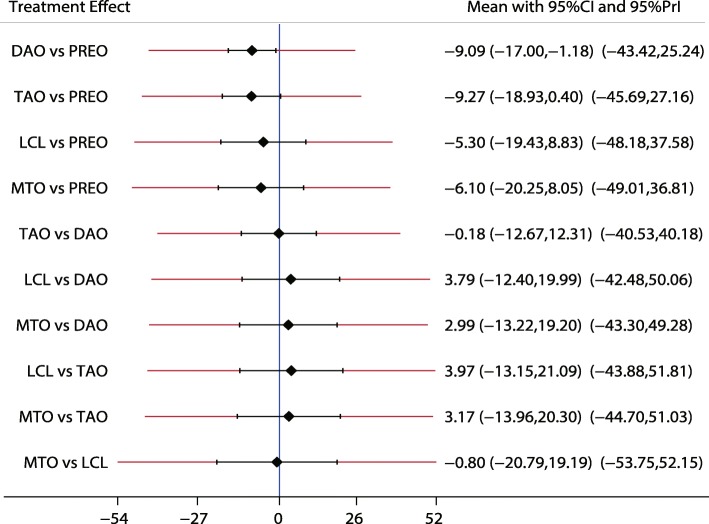
The effect of different treatment strategies based on changes in AP-TC

**Fig. 7:**
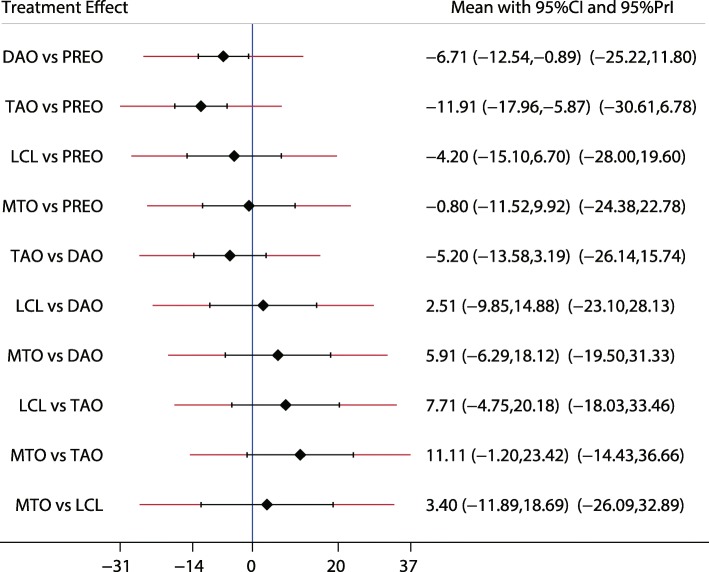
The effect of different treatment strategies based on changes in ALT-TC

**Fig. 8:**
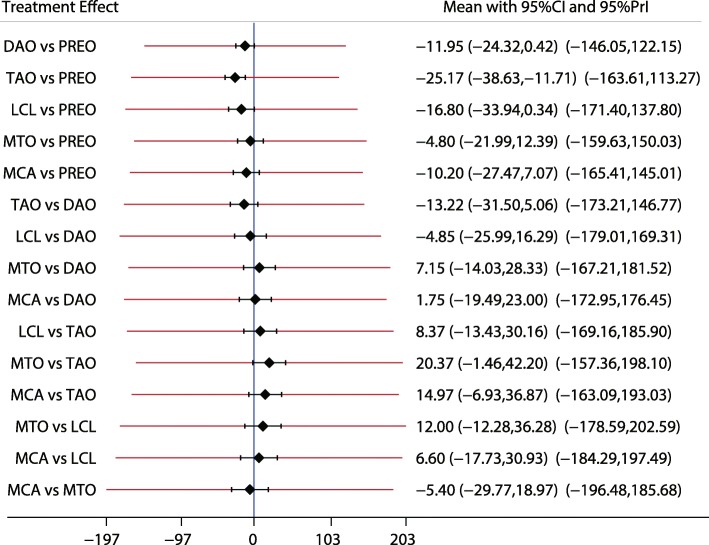
The effect of different treatment strategies based on changes in AP-TNC

**Fig. 9:**
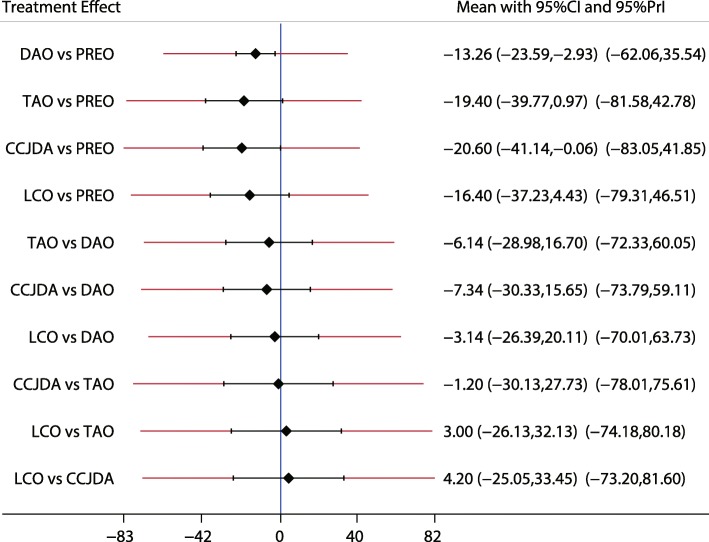
The effect of different treatment strategies based on changes in TNC

**Fig. 10:**
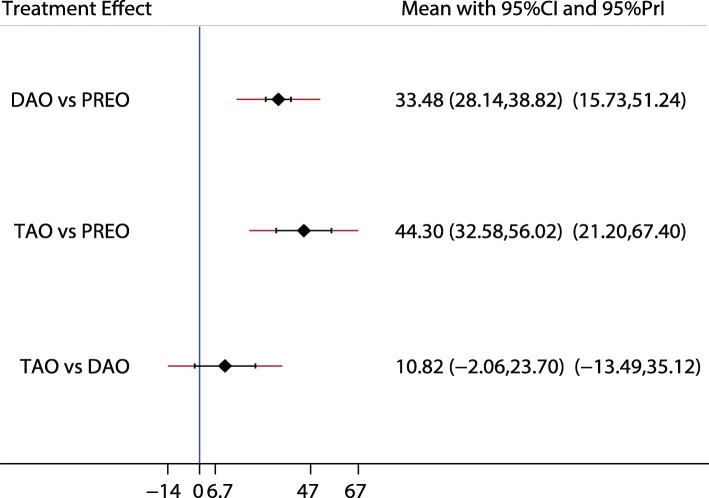
The effect of different treatment strategies based on changes in AOFAS

**Fig. 11:**
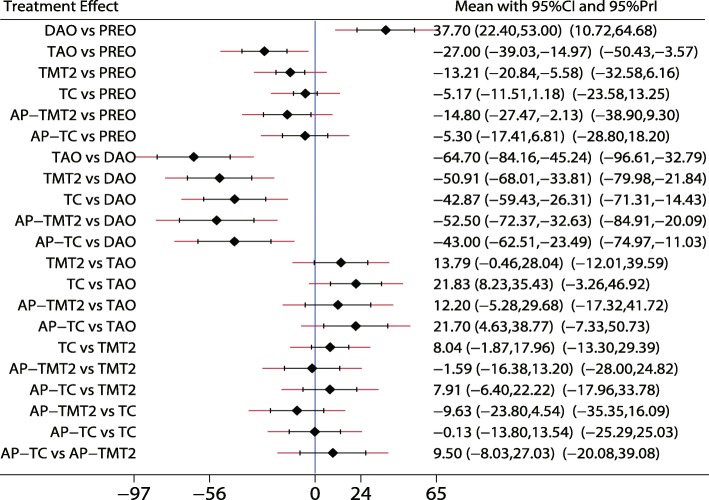
The effect of different treatment strategies based on changes in the function scores

A similar analysis suggested that LCL is the best treatment strategy based on the changes in AP-TMT1 (Fig. 3) and LAT-TMT1 values (Fig. 4). All types of surgical strategies for treating AAFD except for medial translational osteotomy (MTO) are associated with lower AP-TMT1 values (SMD − 2.60; 95%CI − 9.01 to 3.80; Fig. 3). Additionally, the double arthrodesis (DAO), TAO, and LCL showed significant reduction in the levels of LAT-TMT1 as compared with corresponding preoperative values (Fig. 4). Finally, TAO showed the highest probability as the best treatment strategy based on changes in the LAT-arch height (Fig. 5), AP-TC (Fig. 6), ALT-TC (Fig. 7), AP-TNC (Fig. 8), TNC (Fig. 9), AOFAS (Fig. 10), and function scores (Fig. 11).

Both DAO and TAO were associated with higher LAT-arch height values (Fig. 5), increased AOFAS levels (SMD 33.48 and 95%CI 28.14 to 38.82 for DAO; SMD 44.30 and 95%CI 32.58 to 56.02 for TAO; Fig. 10), and lower ALT-TC levels (SMD − 6.71 and 95%CI − 12.54 to − 0.89 for DAO; SMD − 11.91 and 95%CI − 17.96 to − 5.87 for TAO; Fig. 7). Patients who received DAO had significantly lower AP-TC values (SMD − 9.09; 95%CI − 17.00 to − 1.18; Fig. 6), and those who received TAO had significantly reduced levels of AP-TNC (SMD − 25.17; 95%CI − 38.63 to − 11.71; Fig. 8). DAO and CJDA were associated with significantly lower levels of TNC (SMD − 13.26 and 95%CI − 23.59 to − 2.93 for DAO; SMD − 20.60 and 95%CI − 41.14 to − 0.06 for CJDA; Fig. 9). Furthermore, TAO was associated with lower function scores, whereas DAO significantly increased the function scores.

## Discussion

This meta-analysis included 21 studies that evaluated surgical interventions in adult patients with AAFD. Our findings suggested that surgical interventions showed a significant impact on LAT-CP, AP-TMT1, LAT-TMT1, LAT-arch height, AP-TC, LAT-TC, AP-TNC, TNC, AOFAS, and function score. Additionally, we conducted the first network meta-analysis to analyze the effects of different types of surgery for the treatment of AAFD. The results of these analyses indicated that MDCO, LCL, TAO, and DAO might be superior to other surgical approaches for treating AAFD.

The methodological evaluation of each included study was limited by randomization, blinding, allocation concealment, withdrawals and dropouts, and the use of intention-to-treat analysis. In this study, all the included studies were self-contrast trials, and no trial required randomization, blinding, or allocation concealment. Although most of the trials reported withdrawals, dropouts, and the use of intention-to-treat analysis, other biases also contributed to heterogeneity in each study. Ultimately, taking into consideration the unsatisfactory quality of the included studies, we critically provided our recommendations for the treatment of patients with AAFD.

Cao et al. reported that MDCO with reconstruction of the posterior tibial tendon insertion on the navicular bone is considered as an effective treatment for flexible flatfoot with symptomatic accessory navicular and that this method is associated with excellent clinical outcomes and correction of the deformity [30]. Mehta et al. found that MTA showed a reliable and reproducible correction of the deformity seen in rigid stage III posterior tibial tendon dysfunction [33]. Iossi et al. suggested that clinical and radiographic parameters are also important to consider when choosing bony realignment procedures to reconstruct a flexible flatfoot deformity [32]. For the treatment of more severe deformities, LCL resulted in a greater radiographic improvement in alignment. A MDCO alone is also a valuable tool to correct these deformities, although it provides a different level of correction when compared with LCL. Overall, the diverse outcome assessments used in various interventions remains to be the greatest obstacle for the improvement of treatments for AADF, because it is difficult to compare these non-uniform results. Therefore, a systematic review was needed to define the comparison among the surgical approach treatments.

The intervention methods tested in our included studies were restricted to surgical treatments because they are widely used as the first-line treatments in AAFD patients. To increase the reliability of our study, we excluded the studies with high design bias because the treatment and outcome records of AADF, especially the pain and urgency records, are subjected to high subjectivity. We also comparatively analyzed the various surgical treatment approaches by network meta-analysis.

Various surgical types are currently used for AAFD treatment, but there is no widely accepted effective treatment to date. These surgical types include LCL, MTO, TAO, DAO, MCA, MCA, MDCO, excision of accessory navicular with reconstruction of posterior tibial tendon insertion on navicular, FDL, MTA, and reconstruction of the PTT. Our network meta-analysis suggested that among these methods, MDCO, LCL, and TAO might be considered as relatively better surgical strategies for the treatment of patients with AAFD.

The results of our traditional meta-analysis revealed that AAFD patients who received a surgical intervention showed significant improvement with LAT-CP, AP-TMT1, LAT-TMT1, LAT-arch height, AP-TC, LAT-TC, AP-TNC, TNC, AOFAS, and function score values when compared with their preoperative levels. However, several studies reported inconsistent results. Firstly, Iossi et al. showed that a FDL transfer to the navicular and bony realignment showed no significant impact on the function score, whereas the LCL results showed a greater radiographic improvement in alignment [32]. Furthermore, Haeseker et al. suggested that LCL by means of calcaneus osteotomy rather than distraction arthrodesis of the calcaneocuboid joint assists in the correction of stage II posterior tibial tendon dysfunction, but showed no effect on the function score [27]. As there were only few trials reported for each specific surgical strategy, and so we could not conduct a stratified analysis based on the types of surgical intervention. However, we were able to comprehensively compare these methods by performing a network meta-analysis.

Our study has few limitations. Firstly, we did not have access to specific data of individuals for all the trials, and so our statistical analysis could only be performed at a study level. Secondly, there are limited studies using single treatment, which in turn could reduce the reliability of our meta-analysis. Thirdly, there was heterogeneity in most of the outcomes among the included studies, attributing to the non-standardization of outcome assessment. Fourthly, the pre-operative stages of patients might affect the treatment effects of surgical strategies, whereas most of the included studies did not provide these information in detail. Finally, we were not able to use a subgroup analysis and meta-regression to reduce the heterogeneity because there were too few studies using a single surgical intervention for the treatment of AADF. Therefore, unifying the outcome standard is very important for further research to improvise the treatments for AADF.

## Conclusion

The findings of this study suggested that surgical intervention in AADF patients is associated with beneficial prognosis. Furthermore, MDCO, LCL, and TAO are the three surgical strategies that showed the most effective treatments in patients with AAFD. Further research would not only benefit from the addition of well-designed studies, but also from the publication of studies that focused on the pathogenesis and therapeutic mechanisms of AADF, which further improves the understanding of the disease and its treatments.

## Additional file

Additional file 1: PRISMA Checklist. (DOC 72 kb)

## Abbreviations

AAFD: Adult acquired flatfoot deformity
AOFAS: American Orthopedic Foot and Ankle Society
AP-TC: Anteroposterior talocalcaneal
AP-TMT1: Anteroposterior talo-first metatarsal
AP-TNC: Anteroposterior-talonavicular coverage
DAO: Double arthrodesis
FDL: Flexor digitorum longus
LAT-CP: Lateral angle talocalcaneal-calcaneal pitch
LAT-TC: Lateral angle talocalcaneal-talocalcaneal
LCL: Lateral column lengthening
MDCO: Medial displacement calcaneal osteotomy
MTA: Modified triple arthrodesis
SMD: Standardized mean differences
SUCRA: Surface under the cumulative ranking
TAO: Triple arthrodesis
